# Limited sampling strategies for individualized BAX 855 prophylaxis in severe hemophilia A: in silico evaluation

**DOI:** 10.1097/MBC.0000000000001204

**Published:** 2023-04-05

**Authors:** Laura H. Bukkems, Tine M.H.J. Goedhart, C. Michel Zwaan, Marjon H. Cnossen, Ron A.A. Mathôt

**Affiliations:** a Department of Clinical Pharmacology – Hospital Pharmacy, Amsterdam University Medical Centers, Amsterdam; b Department of Pediatric Hematology and Oncology, Erasmus MC Sophia Children's Hospital, University Medical Center Rotterdam, Rotterdam, The Netherlands

**Keywords:** extended half-life, factor VIII, hemophilia A, limited sampling strategy, pharmacokinetics

## Abstract

Limited sampling strategies (LSS) lower the burden of pharmacokinetic (PK)-guided dosing, but an extensive evaluation of LSS for BAX 855 (Adynovi) is currently lacking. This study aimed to develop a LSS for BAX 855 and combine this with a LSS of a standard half-life (SHL) factor VIII (FVIII) concentrate in a clinical setting.

**Methods:**

Individual PK parameters of BAX 855 were estimated for 10 000 virtual patients with severe hemophilia A using Monte Carlo simulations. Several LSS consisting of 2–6 samples were examined based on patient burden, bias and accuracy of clearance, elimination half-life, volume of distribution and trough levels at 72 h (C72). Analyses were performed separately for adults and children <12 years.

**Results:**

The preferred LSS for BAX 855 consisted of three sampling points at 15–30 min, 48 h and 72 h for both adults (mean accuracy C72: 14.0% vs. 10.8% using six samples) and children (mean accuracy C72: 14.9% vs. 11.4% using six samples). The best strategy with two samples (peak, 48 h) resulted in an adequate, but lower accuracy than strategies with ≥3 samples (mean accuracy C72: 22.3%). The optimal combination of the LSS of SHL FVIII and BAX 855 led to six samples during four clinical visits.

**Conclusion:**

This in silico study has identified that two to three samples are necessary to estimate the individual PK of BAX-855 adequately. These samples can be collected in one or two clinical visits. When combining PK profiling of SHL FVIII and BAX 855, six samples during four clinical visits are needed.

## Introduction

Prophylactic treatment with factor VIII (FVIII) concentrates has substantially improved clinical outcomes for patients with hemophilia A [1]. However, a wide inter-individual variability in FVIII pharmacokinetics (PK) is observed after FVIII concentrate administration [2]. Therefore, it is recommended to tailor the FVIII dose to the individual patient by so called ‘PK-guided dosing’ [3].

To characterize the PK of an individual patient, several blood samples are collected after FVIII concentrate administration; a so called ‘PK profile’. Historically, at least 10 blood samples after an infusion were collected [4]. However, this intensive sampling schedule is unsuitable for clinical practice, due to time investment and burdening of the patient, especially when children are concerned. As currently recommended in guidelines, individual PK parameters can also be derived using Bayesian estimation [3,5,6]. This technique combines information from a population PK model with data from the individual patient, as a result only two to five FVIII levels are necessary to adequately estimate individual PK parameters [7–13].

Patients with hemophilia A may need to switch between FVIII concentrates, either as a consequence of the availability of new factor concentrates, for instance from standard half-life (SHL) to an extended half-life (EHL) concentrate, or due to price agreements. During this switch, PK-guided dosing can aid in determining the adequate dose of the new concentrate for each individual patient [13]. Often, individual PK parameters are determined for the new concentrate. During our clinical experience, we observed that it may also be beneficial to determine the individual PK parameters of the currently used factor concentrate to estimate FVIII levels during bleeds, physical activities and FVIII trough and peak levels, as these FVIII levels are informative to set target factor levels for the PK-guided dosing of the new concentrate [14]. Yu *et al.*[15] also describe knowing the individual PK parameters of the currently used concentrate as an ideal scenario. Of course, the extra blood sampling necessary to perform PK profiling with both the current and new concentrate is a disadvantage, obligating the patient to more hospital visits than when only the individual PK of the new concentrate is determined.

However, this number of blood samples can be reduced, when limited sampling strategies (LSS) for both concentrates are concomitantly applied. For several SHL and EHL FVIII concentrates LSS have already been published [7–12,16]. Hajducek *et al.*[16] evaluated seven LSS for Adynovi and reported the error in the terminal half-life. However, extensive evaluation of a LSS for BAX 855 (Adynovi) with more sampling times including evaluation of other interesting PK parameters and evaluation of sampling schemes for children separately are still lacking in literature. Therefore, the aim of this study was to develop a LSS for BAX 855 for adults and children and subsequently to illustrate how LSSs of two different factor concentrates can be combined, resulting in fewer blood samples and clinical visits.

## Materials and methods

### Simulated patients

To develop a LSS for BAX 855 (Adynovi/Adynovate, Takeda Pharmaceutical Company Limited, Japan), Monte Carlo simulations were performed for 10 000 virtual patients with severe hemophilia A in NONMEM. The Monte Carlo method takes repetitively random samples from the distribution of PK parameters described by a population PK model. The method also takes unexplained inter-individual variability into account, resulting in different PK parameters for individuals with similar patient characteristics. For this analysis, the published population PK model of BAX 855 from the WAPPS-hemo database based on one-stage assay FVIII levels was used [17]. This model was a two compartment model in which clearance was depended on the fat-free mass and age of the patient and central volume of distribution on fat-free mass. A virtual patient population was created with R software (v3.5.2) so that the patients’ age, body weight and fat-free mass resembled the patient population used for model building. Individual PK parameters [clearance (CL), steady state volume of distribution (VSS) and elimination half-life (*t*_1/2_)] were obtained for this virtual patient population and FVIII level over time profiles were constructed. For simplicity, it was assumed that all patients had severe hemophilia A and had no endogenous baseline FVIII level. A steady-state dose of 50 IU/kg every 72 h was administrated to the virtual patients and the dose was rounded to the nearest dose of 250 IU, accounting for the available vial sizes.

### Limited sampling strategy BAX 855

Several LSS including 2–6 samples were developed based on the six time points (15–30 min, 4, 24, 48, 72 and 96 h after concentrate infusion) reported in the practical proposal for switching from SHL to EHL concentrates from Nederlof *et al.*[14] We focused on LSS including at least one peak level and one other level (Table 1). In clinical practice, it is unlikely that sampling will be performed on the exact time points. Therefore, we applied a window of 1 h around the 4-h sampling time and a window of 4 h around the 24, 48, 72 and 96 h time points. Importantly, we do not describe predose levels as sample point, since the predose and the latest sampling time point are similar when the sampling is efficiently timed and the previous doses are known (later described in more detail). The M3 method was used to handle samples below the limit of quantification (BQL; <0.01 IU/ml). This method estimates the likelihood that the sample is BQL [18].

**Table 1:** Evaluated limited sampling strategies (LSS) for BAX 855. LSS11 and LSS12 include two samples around 48 h or 72 h. LSS7 (in green) was selected as preferred strategy

Time point	15–30 min	4 h	24 h	48 h	72 h	96 h
Six samples						
LSS1	X	X	X	X	X	X
Four samples						
LSS2	X		X	X	X	
LSS3	X		X	X		X
LSS4	X		X		X	X
LSS5	X			X	X	X
Three samples						
LSS6	X		X	X		
LSS7	X			X	X	
LSS8	X				X	X
LSS9	X		X		X	
LSS10	X		X			X
LSS11	X			XX		
LSS12	X				XX	
Two samples						
LSS13	X		X			
LSS14	X			X		
LSS15	X				X	
LSS16	X					X

The results of each LSS were assessed by calculation of the relative error for the PK parameters (CL, V1, *t*_1/2_) and the estimated trough level at 72 h (C_72_) for BAX855 or estimated trough level at 48 h (C_48_) for SHL FVIII [Eq. (1)]. The relative error was visualized in boxplots. The bias calculated by the mean percentage error [MPE, Eq. (2)] and accuracy calculated by a mean absolute percentage error [MAPE, Eq. (3)] of the examined LSSs were determined and LSSs with a MPE value outside −5% to 5% and MAPE >25% were deemed inadequate, such as also applied in other published limited sampling strategy studies [19,20].

(1) $$\text{relative}\;\text{error}\;=\;\frac{\theta i_{j}-\theta_{\text{true},i}}{\theta_{\text{true},i}}\times 100\%$$

(2) $$\text{MPE}\;=\;\frac{1}{n}\;\sum_{i=1}^{n}\left(\text{relative}\;\text{error}\right)$$

(3) $$\text{MAPE}\;=\;\frac{1}{n}\;\sum_{i|=1}^{n}\left|\;\text{relative}\;\text{error}\right|$$

In which $\theta_{ij}$ describes the individual PK parameter for the *i*th individual with the *j*th LSS set and the *θ*_true,i_ the true PK parameter obtained by Monte Carlo simulation for the *i*th individual.

In general, children have a higher FVIII clearance per kg body weight [21,22]. Therefore, the adequacy of the LSSs was evaluated separately for adults and children <12 years.

### Limited sampling strategy standard half-life factor VIII

To lower the burden for the patient, we compared the LSS sampling strategy for SHL FVIII concentrates after a steady state dose of 40 IU/kg every 48 h including a 15–30 min, 4 h, 24 h and 48 h sample to a strategy without the 4 h sample using the same virtual population of 10 000 patients [7,14]. The published population PK model of Bjorkman was used and for all patients’ administration of a recombinant SHL FVIII concentrate was assumed [21]. Further methods were similar to the methods described for LSS of BAX 855.

### Obtaining standard half-life and extended half-life pharmacokinetics profiles

Subsequently, the LSS showing the best predictive performance, indicated by lowest bias, highest accuracy and lowest patient burden, was selected as PK profile strategy for BAX 855. This strategy was combined with the selected LSS of SHL FVIII concentrates, to illustrate how PK profiles of both SHL FVIII and BAX 855 can be obtained in a clinical setting efficiently.

## Results

### Simulations

**Fig. 1:**
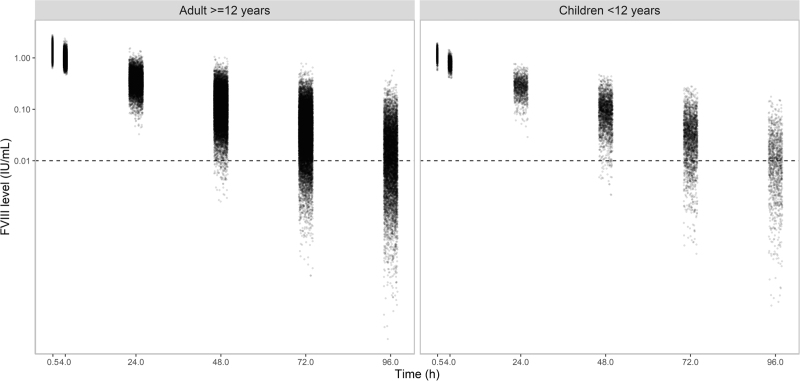
FVIII levels of 10 000 simulated patients for limited sampling strategy analysis. The left figure depicts the FVIII levels for adults (≥12 years, 8833 virtual patients) and the right figure for children <12 years (1167 virtual patients). The dotted horizontal line represents the lower limit of quantification (LLOQ, 0.01 IU/ml). For 9% and 43% of the adults the FVIII level was below the quantification limit (BQL) 70–74 h and 94–98 h after administration, respectively. For children, 12% and 52% of the FVIII levels were BQL 70–74 and 94–98 after administration, respectively. FVIII, factor VIII.

To evaluate several LSS, a patient population was simulated with characteristics similar to the population used for development of the BAX 855 population PK model (Table 2) [17]. In Figure 1, Supplemental Digital Content, correlations between the patient characteristics are given. The simulated FVIII levels used for the LSS analysis are shown in Fig. 1. For 9% of the adults the FVIII level was BQL (<0.01 IU/ml) 70–74 h after administration. In addition, 43% had a FVIII level BQL 94–98 h after administration. For children, 12% and 52% of the FVIII levels were BQL 70–74 and 94–98 after administration, respectively.

**Table 2:** Characteristics of the simulated population

	Original population used for population model	Simulated population		
		Total	Adults	Children <12 years
*N*	154	10 000	8833	1167
Age (years)	19.0 (3.4–72.8)	24.3 (3.0–72.0)	26.2 (12.0–72.0)	9.3 (3.0–11.9)
Body weight (kg)	70.0 (14.8–150.0)	70.7 (14.8–149.9)	74.3 (23.5–149.9)	39.8 (14.7–81.9)
BMI (kg/m^2^)	23.2 (13.5–50.0)	23.7 (10.4–51.1)	24.4 (12.9–51.1)	19.6 (10.4–33.7)
Fat free mass (kg)	−	55.6 (12.8–97.8)	57.5 (18.3–97.8)	34.5 (12.8–64.8)

Data is presented as median (range) or number.

### Limited sampling strategies BAX 855

**Fig. 2:**
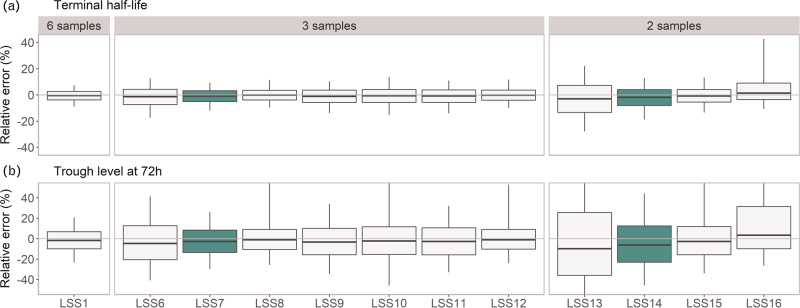
Relative error between the *true* simulated PK parameters and PK parameters estimated by Bayesian analysis for a selection of the evaluated limited sampling strategies of BAX 855 for adults (≥12 years). The parameters elimination half-life (a) and FVIII level at 72 h after dose (b) are presented. The boxes of the boxplots present the median (middle line) and interquartile range (IQR) and the whiskers extend to the 5th and 95th percentile. The green boxes present the preferred limited sampling strategies (LSS7 and LSS14) with the smallest relative errors. For readability, the y-axis of the C_72_ plots are limited from −50% to 50%, while maintaining all data. PK, pharmacokinetics.

Figure 2 and Table 3 show the results of the evaluation of several LSS for BAX 855 for patients ≥12 years. As can be seen in Fig. 2, some strategies performed better than others. The following strategies per number of sample points resulted in the lowest bias and least inaccuracy: LSS5 (peak, 48, 72, 96 h) using four samples, LSS7 (peak, 48, 72 h) using three samples and LSS14 (peak, 48 h) or LSS15 (peak, 72 h) using two samples. The relative error observed in the examined LSSs including four samples were rather similar and all evaluated strategies met the prespecified criteria. Taking both the burden for the patient and the results of our evaluation, we considered LSS7 (15–30 min, 48 h, 72 h) with three samples as the best strategy. If a patient has already been switched to BAX 855, the pre dose level of PK profile can be timed 72 h after home infusion. This way, samples for the BAX 855 PK profile can be obtained in only two visits to the clinic. When inspecting the relative errors of the LSSs including two samples, the lowest errors in clearance, volume of distribution and terminal half-life are observed for LSS15 (peak, 72 h), closely followed by LSS14 (peak, 48 h). However, the relative error of the C_72_ of LSS15 was >25%, presumably caused by BQL samples. Therefore, we selected LSS14 as the preferred strategy when two samples are taken. This LSS had a higher bias and lower accuracy than strategies with three or more samples. Namely, the MPE of C_72_ was −4.0% [95% confidence interval (CI): −4.7 to −3.4] and the MAPE 22.3% (95% CI: 21.8 to 22.8) for LSS14 (two samples), whereas these were −1.9% (95% CI: −2.4 to −1.4) and 14.0% (95% CI: 13.7–14.4) for LSS7 (three samples) and −1.3% (95% CI: −1.6 to −1.0) and 10.8% (95% CI: 10.6 to 11.0) for LS1 (six samples), respectively (Table 3).

**Table 3:** Bias and inaccuracy of evaluated parameters and examined limited sampling strategies of BAX 855 for adults (≥12 years), represented by the mean percentage error (MPE) and mean absolute percentage error (MAPE) including 95% confidence interval, respectively

	Clearance (CL)		Steady state distribution volume (*V*_ss_)		Terminal half-life (*t*_1/2_)		Predicted FVIII level at *t* = 72 h (C_72_)	
	MPE (%)	MAPE (%)	MPE (%)	MAPE (%)	MPE (%)	MAPE (%)	MPE (%)	MAPE (%)
Six samples								
LSS1	1.3 (1.2–1.5)	5.9 (5.8–6.0)	0.9 (0.7–1.1)	7.9 (7.8–8.1)	−0.6 (−0.7 to −0.5)	3.9 (3.8–4.0)	−1.3 (−1.6 to −1.0)	10.8 (10.6–11.0)
Four samples								
LSS2	1.7 (1.5–1.9)	6.7 (6.6–6.9)	0.9 (0.7–1.1)	9.1 (9.0–9.3)	−0.9 (−1.0 to −0.8)	5.0 (4.9–5.1)	−1.9 (−2.3 to −1.6)	13.1 (12.9–13.4)
LSS3	1.8 (1.6–2.0)	6.9 (6.8–7.1)	1.0 (0.7–1.2)	9.0 (8.8–9.1)	−0.9 (−1.0 to −0.8)	4.9 (4.8–5.0)	−1.7 (−2.1 to −1.3)	14.2 (13.9–14.4)
LSS4	1.6 (1.4–1.8)	7.1 (7.0–7.2)	1.2 (0.9–1.4)	9.0 (8.8–9.1)	−0.6 (−0.7 to −0.4)	4.5 (4.4–4.6)	−0.1 (−0.6 to −0.3)	13.9 (13.5–14.3)
LSS5	1.6 (1.4–1.8)	7.1 (7.0–7.3)	1.2 (0.9–1.4)	9.3 (9.2–9.5)	−0.6 (−0.7 to −0.5)	4.1 (4.0–4.2)	−1.1 (−1.6 to −0.7)	11.5 (11.2–11.9)
Three samples								
LSS6	2.7 (2.5–2.9)	7.8 (7.6–7.9)	0.8 (0.5–1.0)	9.4 (9.3–9.6)	−1.7 (−1.9 to −1.5)	7.2 (7.1–7.4)	−2.8 (−3.4 to −2.3)	20.2 (19.9–20.6)
LSS7	2.0 (1.8–2.2)	7.4 (7.3–7.5)	1.1 (0.8–1.3)	9.5 (9.4–9.7)	−1.0 (−1.1 to −0.8)	5.1 (5.0–5.2)	−1.9 (−2.4 to −1.4)	14.0 (13.7–14.4)
LSS8	0.9 (0.6–1.1)	8.3 (8.1–8.4)	1.3 (1.1–1.6)	9.5 (9.4–9.7)	0.5 (0.4–0.7)	5.2 (5.1–5.3)	17.3 (13.1–21.5)	30.1 (25.9–34.3)
LSS9	2.2 (2.0–2.4)	7.6 (7.4–7.7)	1.0 (0.7–1.2)	9.1 (9.0–9.3)	−1.2 (−1.4 to −1.1)	5.8 (5.7–5.9)	−1.4 (−2.0 to −0.9)	17.3 (16.9–17.7)
LSS10	2.3 (2.1–2.5)	8.3 (8.2–8.5)	1.4 (1.1–1.6)	9.2 (9.0–9.3)	−0.7 (−0.9 to −0.5)	6.5 (6.4–6.7)	2.4 (1.6–3.2)	23.0 (22.4–23.7)
LSS11	2.1 (1.9–2.3)	7.4 (7.2–7.5)	1.1 (0.8–1.3)	9.6 (9.4–9.8)	−1.0 (−1.2 to −0.9)	5.9 (5.8–6.0)	−1.7 (−2.2 to −1.2)	16.1 (15.8–16.5)
LSS12	0.8 (0.6–1.0)	8.0 (7.8–8.1)	1.3 (1.0–1.5)	9.6 (9.5–9.8)	0.5 (0.3–0.7)	5.3 (5.2–5.5)	16.7 (12.6–20.8)	28.8 (24.8–32.8)
Two samples								
LSS13	5.6 (5.3–6.0)	12.3 (12.0–12.5)	0.9 (0.6–1.1)	9.7 (9.5–9.8)	−2.9 (−3.3 to −2.6)	12.3 (12.1–12.5)	0.4 (−0.7–1.5)	39.1 (38.4–39.8)
LSS14	3.7 (3.5–3.9)	9.1 (8.9–9.1)	1.0 (0.7–1.3)	9.6 (9.5–9.8)	−2.2 (−2.4 to −2.0)	7.6 (7.5–7.7)	−4.0 (−4.7 to −3.4)	22.3 (21.8–22.8)
LSS15	1.8 (1.5–2.0)	8.9 (8.8–9.1)	1.2 (1.0–1.5)	9.6 (9.4–9.7)	−0.2 (−0.4 to −0.1)	6.6 (6.4–6.7)	15.6 (11.4–19.8)	33.8 (29.7–38.0)
LSS16	−3.0 (−3.3 to −2.7)	11.4 (11.2–11.6)	1.7 (1.5–2.0)	9.6 (9.5–9.8)	6.3 (6.0–6.7)	10.9 (10.6–11.2)	97.8 (84.4–111.3)	109.9 (96.5–123.3)

For children a higher percentage of FVIII levels was BQL (52% vs. 43% 94–98 h after administration for patients <12 years vs. ≥12 years). Therefore, the limited sampling strategies were also evaluated separately for children <12 years old. Despite the higher number of BQL samples, the preferred LSS with the lowest bias and best accuracy was the same as for adults (Figure 2 and Table 1, Supplemental Digital Content). For the C_72_ of LSS7 (peak, 48, 72 h) a MPE of −1.1% (95% CI: −2.6 to 0.5) and MAPE of 14.9% (95% CI: 13.6 to 16.2) was observed in comparison to a MPE of −0.7% (95% CI: −1.7 to 0.2) and MAPE of 11.4% (95% CI: 10.7 to 12.1) in the sampling strategy with six samples. The bias (−2.8%, 95% CI: −4.7 to −0.9) and accuracy (22.9%, 95% CI: 21.5 to 24.3) of the two samples design (peak, 48 h) for children was similar to adults.

### Limited sampling strategies standard half-life factor VIII

**Fig. 3:**
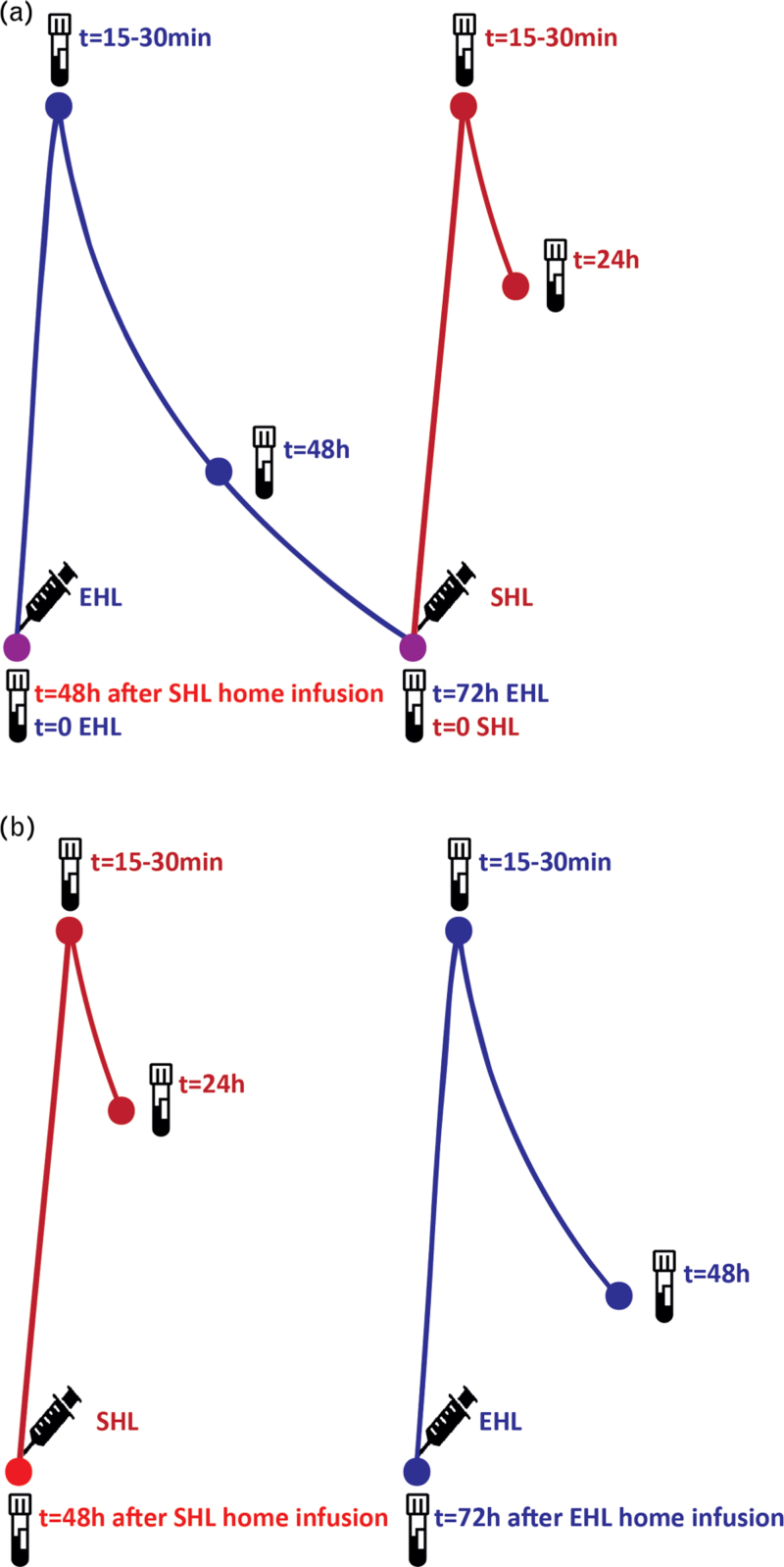
Limited sampling PK profile of SHL FVIII and BAX 855 combined, resulting in the fewest visits to the clinic. The blue color indicates dosing and sampling points of the extended half-life concentrate, while the red color refers to the standard half-life concentrate. Figure 3A demonstrates the combination of PK profiling of both the SHL-FVIII concentrate and BAX855. Figure 3B demonstrates PK profiling separately, when the first sample is taken at a set time point after home infusion of the factor concentrates. FVIII, factor VIII; PK, pharmacokinetics; SHL, standard half-life.

Figure 3, Supplemental Digital Content depicts the results of the evaluation of the LSS for SHL FVIII concentrates. No clinically relevant differences can be seen when the strategy with and without the 4 h sample are compared. Hence, the strategy without the 4 h sample is recommended because of the lower burden for the patient.

### Obtaining standard half-life and extended half-life pharmacokinetics profiles

Figure 3 A illustrates how PK profiling of both the SHL-FVIII concentrate and BAX 855 can be efficiently performed. By sampling the FVIII level before BAX 855 administration and 48 h after home infusion of SHL, the number of samples and consequently the number of visits can be reduced. Seventy-two hours after the EHL administration, one dose of SHL FVIII is administrated, to collect the peak and 24 h samples of the SHL profile. Importantly, when using such method information about prior doses (date, time, dose) is necessary. Furthermore, before estimation of the individual PK parameters on the concentrates, it will be necessary to correct for the residual level of the previous concentrate. This can be handled by using first-order elimination of the residual level or using the described doses and calculated individual PK parameters [23–25]. A limitation of this is that this correction assumes that factor concentrates are measured similarly with the same hemostatic assay. A second possibility is to sample the SHL and EHL curve separately as demonstrated in Fig. 3B. In this manner, correction for a residual level of the previous concentrate is not necessary. However, a disadvantage is that when the patient prefers to administrate the first dose of the EHL concentrate in the clinic, an extra visit is necessary.

## Discussion

In this study, a LSS for the EHL-FVIII concentrate BAX 855 was developed for patients with severe hemophilia A. The best LSSs included sampling time points at 15–30 min, 48 h and 72 h (LSS7) or 15–30 min, 48 h (LSS14) for both adults and children. The blood samples of LSS7 can be obtained during two visits to the clinic, when the 72 h sample is taken as a predose level. In this way - during the first clinical visit - a sample is taken 72 h after BAX 855 is administrated at home, a new BAX 855 dose is administrated and 15–30 min after dose the second sample is collected. Two days later the second clinical visit is planned to take the sample 48 h after the dose.

The selected strategy with two samples (peak, 48 h) resulted in adequate, but lower accuracy than strategies with ≥3 samples. We recommend this two-sample design – which can be collected in one clinical visit – especially for children with difficult venous access or in low-income countries. The two-sample design including a peak and 72 h sample (LSS15) also demonstrated adequate relative errors in the PK parameters. However, for 9% of the adults and 12% of the children the 72 h sample will be BQL and thus undetectable, complicating adequate estimation of the 72 h trough level for these individuals when this LSS is applied. Treatment centers can decide to select LSS15, but for 9–12% of the patients, it necessitates taking a new 72 h sample when the sample is BQL.

Interestingly, similar LSS for both adults and children were selected, despite a difference in the number of BQL samples. This suggests that the difference in BQL samples is too small (9% vs. 12% for the 72 h sample) to result in better performance of other sampling schemes. Moreover, in our results, the LSS including a 96 h sample, performed less adequate than sample schemes including 48 or 72 h samples. This is caused by a high percentage (45%) of patients for who this sample is BQL and thus less informative. Presumably, this sample can be informative for patients when being above BQL. However, with the current approach beforehand it is not known for which patients this will be the case. A possible solution would be to use personalized limited sampling approaches [26].

In our study, we also illustrated how the LSSs of SHL FVIII concentrate and BAX 855 can be combined resulting in six blood samples during four visits. In contrast, when the two PK profiles are not combined and especially when the predose level is not used as trough level, eight samples during six visits to the clinic are needed to construct both PK profiles. Importantly, the approach of combining the sampling of two PK profiles may be applied to other FVIII and FIX concentrates, when limited sampling strategies of the specific factor concentrate products are present, such as for rFVIIIFc (Elocta). When this combined sample design for two concentrates is performed, individual PK parameters for both concentrates can be obtained. Using Bayesian estimation, knowledge about previous FVIII levels during onset of bleeds and physical activities can be obtained when – in for instance a logbook of a patient - administration times of factor concentrate doses are available. These levels can be informative to set target levels for the PK-guided dosing of the new concentrate. However, it is important to bear three limitations in mind. Firstly, the PK–pharmacodynamics (PD) relation between two factor concentrates may be different, meaning that a similar targeted FVIII level could result in a different effect and thus different bleeding tendency. Previously published studies are too limited to compare the PK–PD relation of factor concentrates, but differences in obtained annual bleeding rate (ABR) values with similar doses could indicate a different PK–PD relation. Secondly, time spent above or below certain factor level may be clinically relevant and differs between for instance SHL and EHL factor concentrates, although an identical trough level is targeted [5]. To overcome this limitation, the time above a certain factor level could also be calculated. Thirdly, differences in one-stage and chromogenic assay can vary per factor concentrate, making it more difficult to compare the factor levels on both concentrates [27].

In the preferred strategy for BAX 855, the peak, 48 h and 72 h sample were present. These results differ from Hajducek's preferred LSS based on *t*_1/2_ estimations of BAX 855, namely predose, 2 h, 24 h and 72 h (four samples) or predose, 2 h and 72 h (three samples) [16]. Our most comparable strategies are LSS9 and LSS15, respectively, with the exception that we included a peak sample instead of a 2 h sample. Only looking at the *t*_1/2_ estimation, in line with Hajducek's methodology, LSS15 indeed seemed the best three-sample strategy (when the 72 h level is taken as a predose level). However, using LSS15, C_72_ could not be estimated accurately. Therefore, we recommend to use our preferred LSS as sampling a peak factor level is more convenient than sampling a 2-h sample. Our finding is partly consistent with the LSS for rFVIIIFc (Elocta) of Mc Enemy-King *et al.*, which selected a 72 h trough, 1 h peak and 72 h as 3-point sample design for adults [12]. Our results cannot be compared to a LSS for BAY 94–9027 (Jivi) as Solms *et al.*[28] only evaluated the following sampling designs in their study: one sample, two samples (4 h, 48 h) or three samples (4 h, 24 h, 48 h). The selected LSS for BAX 855 includes two to three samples. This is in accordance with the recommendation of the subcommittee on FVIII, factor XI and rare bleeding disorders of the ISTH that proposed two to four post infusion samples [13]. Furthermore, the study of Blanchette *et al.*[29] compared a 6-sample design to a two-sample design in a PK study of Advate with 39 patients with hemophilia A, and concluded that the 2-sample design showed sufficient accuracy to be used in routine clinical care. This conclusion was based on an substantial and almost perfect interclass correlation for clearance (0.73) and *t*_1/2_ (0.84) between the PK parameters in the six- and two-sample design. Though, it should be noted that the PK parameters calculated with a six-sample design are not the exact (true) individual PK parameters, making the agreement of the two-sample design with true individual PK parameters smaller.

The results of this study are based on simulations and therefore have not yet been validated with real-life patient data in a clinical setting. However, PK data collected by the Web-Accessible Population Pharmacokinetic Service (WAPPS) could be used.

Furthermore, the predictive performance of the LSSs is dependent on the population PK model that is used for the simulations and may differ depending on the patient characteristics and sampling design of the data used for model development. The population PK model of BAX 855 used in this analysis included 154 hemophilia A patients from 3 to 72 years [17]. The results therefore reflect a population that is similar to the population that was used for building this model.

## Conclusion

This in silico study has identified that two to three samples are necessary to estimate the individual PK of BAX-855 adequately. Sampling a peak (15–30 min), 48 h and 72 h resulted in the best predictive performance for the PK of BAX-855. These three samples can be collected within two visits to the clinic, when the 72 h sample is taken as predose level 72 h after BAX 855 home infusion. The best 2-sample design with samples at 15–30 min and 48 h, can be collected within one visit to the clinic. Moreover, we illustrated how the PK profile of a current and new concentrate can be combined. This approach leads to fewer clinical visits, whereas concomitantly collecting valuable information on individual PK parameters of the currently used concentrate. These PK parameters can be used to estimate FVIII levels during previous bleeds, physical activities and FVIII trough and peak levels, which are helpful to better set target levels for the PK-guided dosing of the new concentrate.

## Acknowledgements

Funding and acknowledgments: This study was performed as part of the OPTI-CLOT international multicenter research consortium, ‘Patient tailOred PharmacokineTIc-guided dosing of CLOTting factor concentrates and desmopressin in bleeding disorders,’ which is currently WP6 within the SYMPHONY consortium. L.H.B. and M.H.J.G. are funded by the SYMPHONY consortium. The SYMPHONY consortium aims to orchestrate personalized treatment in patients with bleeding disorders, and is a unique collaboration between patients, healthcare professionals and translational & fundamental researchers specialized in inherited bleeding disorders, as well as experts from multiple disciplines. It aims to identify best treatment choice for each individual based on bleeding phenotype. In order to achieve this goal, work packages have been organized according to three themes e.g. Diagnostics (workpackage 3&4); Treatment (workpackages 5–9) and Fundamental Research (workpackages 10–12). This research received funding from the Netherlands Organization for Scientific Research (NWO) in the framework of the NWA-ORC Call grant agreement NWA.1160.18.038. Principal investigator: Dr M.H. Cnossen; project coordinator: Dr S.H. Reitsma.

Beneficiaries of the SYMPHONY consortium: Erasmus University Medical Center-Sophia Children's Hospital, project leadership and coordination; Sanquin Diagnostics; Sanquin Research; Amsterdam University Medical Centers; University Medical Center Groningen; University Medical Center Utrecht; Leiden University Medical Center; Radboud University Medical Center; Netherlands Society of Hemophilia Patients (NVHP); Netherlands Society for Thrombosis and Hemostasis (NVTH); Bayer B.V., CSL Behring B.V., Swedish Orphan Biovitrum (Belgium) BVBA/SPRL.

Autorship contributions: L.H.B., M.H.J.G. and R.A.A.M. performed the analysis. C.M.Z. and M.H.C. gave clinical input. L.H.B. and M.H.J.G. wrote the manuscript. M.H.C. and R.A.A.M. supervised the study. All authors contributed substantially to the critical revision of the manuscript and approved the final draft.

Availability of data and materials: Requests to access the data collected in the OPTI-CLOT trial should be sent to the corresponding author.

### Conflicts of interest

M.H.C.'s institution has received investigator-initiated research and travel grants as well as speaker fees over the years from the Netherlands Organisation for Scientific Research (NWO) and Netherlands National research Agenda (NWA), the Netherlands Organization for Health Research and Development (ZonMw), the Dutch Innovatiefonds Zorgverzekeraars, Baxter/Baxalta/Shire/ Takeda, Pfizer, Bayer Schering Pharma, CSL Behring, Sobi Biogen, Novo Nordisk, Novartis and Nordic Pharma, and for serving as a steering board member for Roche, Bayer and Novartis for which fees go to the Erasmus MC as an institution. R.A.A.M. has received grants from governmental and societal research institutes such as NWO, ZonMW, Dutch Kidney Foundation and Innovation Fund and unrestricted investigator research grants from Baxter/ Baxalta/ Shire/Takeda, Bayer, CSL Behring, Sobi and CelltrionHC. He has served as advisor for Bayer, CSL Behring, Merck Sharp & Dohme, Baxter/ Baxalta/ Shire/Takeda. All grants and fees paid to the institution. Other authors have no conflict of interest to declare for this paper.

## Supplementary Material

Supplemental Digital Content
