# Supplementary material for: Limited sampling strategies for individualized BAX 855 prophylaxis in severe hemophilia A: in silico evaluation
Source: Blood Coagul Fibrinolysis. 2023 Apr 5;34(3):171–8. doi: 10.1097/MBC.0000000000001204 (PMC10101132; doi:10.1097/MBC.0000000000001204)
Supplement: Supplemental Digital Content [file blcof-34-171-s001.docx]

**SUPPLEMENTARY FILES of**

**Limited sampling strategies for individualized BAX 855 prophylaxis in severe hemophilia A: in silico evaluation**

L.H. Bukkems^1*^, M.H.J. Goedhart^2*^, C.M. Zwaan^2^, M.H. Cnossen^2*^ & R.A.A Mathôt^1*^, for the OPTI-CLOT study group and SYMPHONY consortium.

^1^Department of Clinical Pharmacology - Hospital Pharmacy, Amsterdam University Medical Centers, Amsterdam, The Netherlands; ^2^Department of Pediatric Hematology and Oncology, Erasmus MC Sophia Children’s Hospital, University Medical Center Rotterdam, Rotterdam, the Netherlands

*Shared first-author and last authors


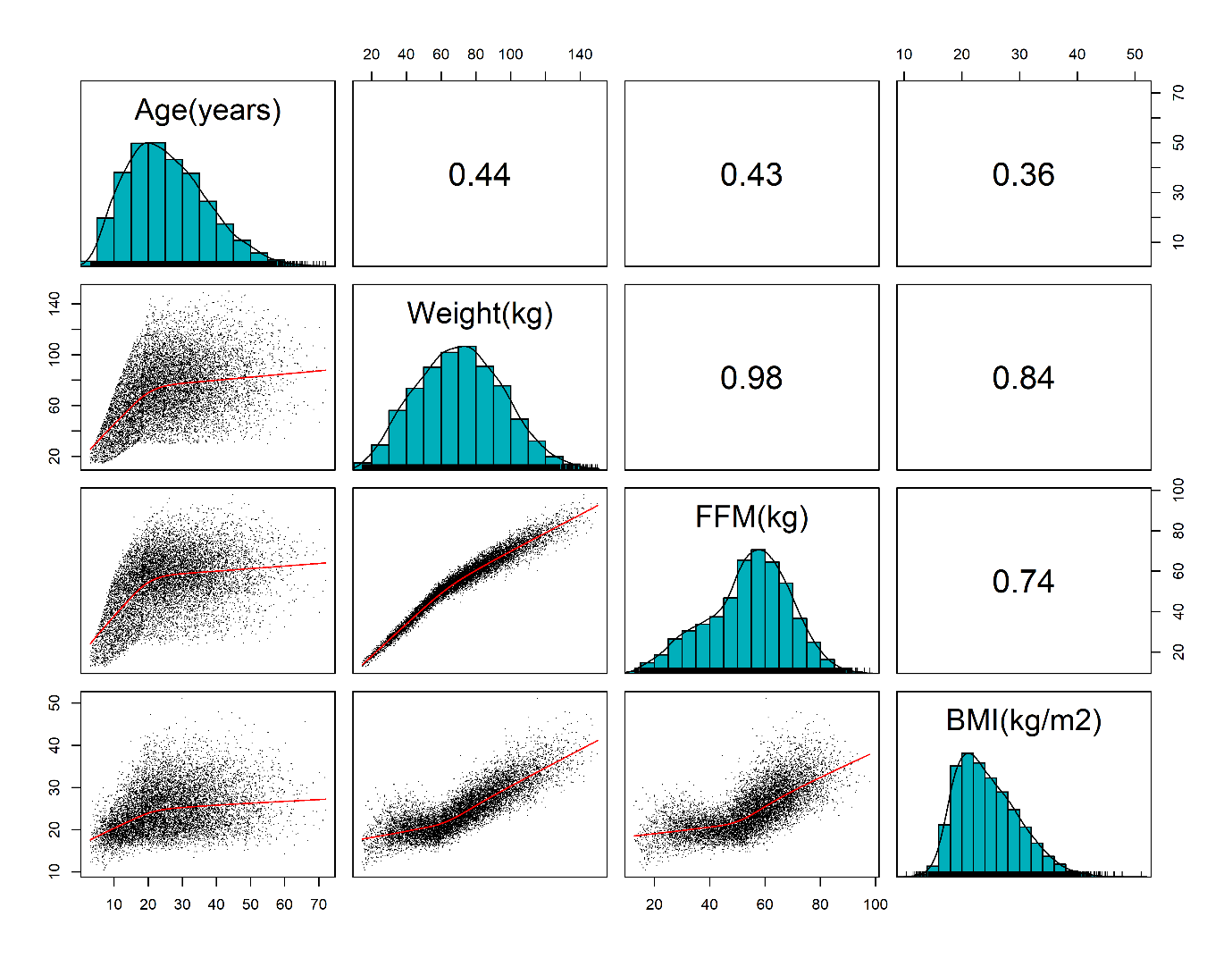


**Figure 1: Correlation between patient characteristics of the simulated population**. The diagonal depicts histograms with density plots of the patient characteristics. The lower off diagonal presents bivariate scatter plots. Above the diagonal the Pearson correlation coefficients are included. FFM: fat-free mass, BMI: body mass index.

**
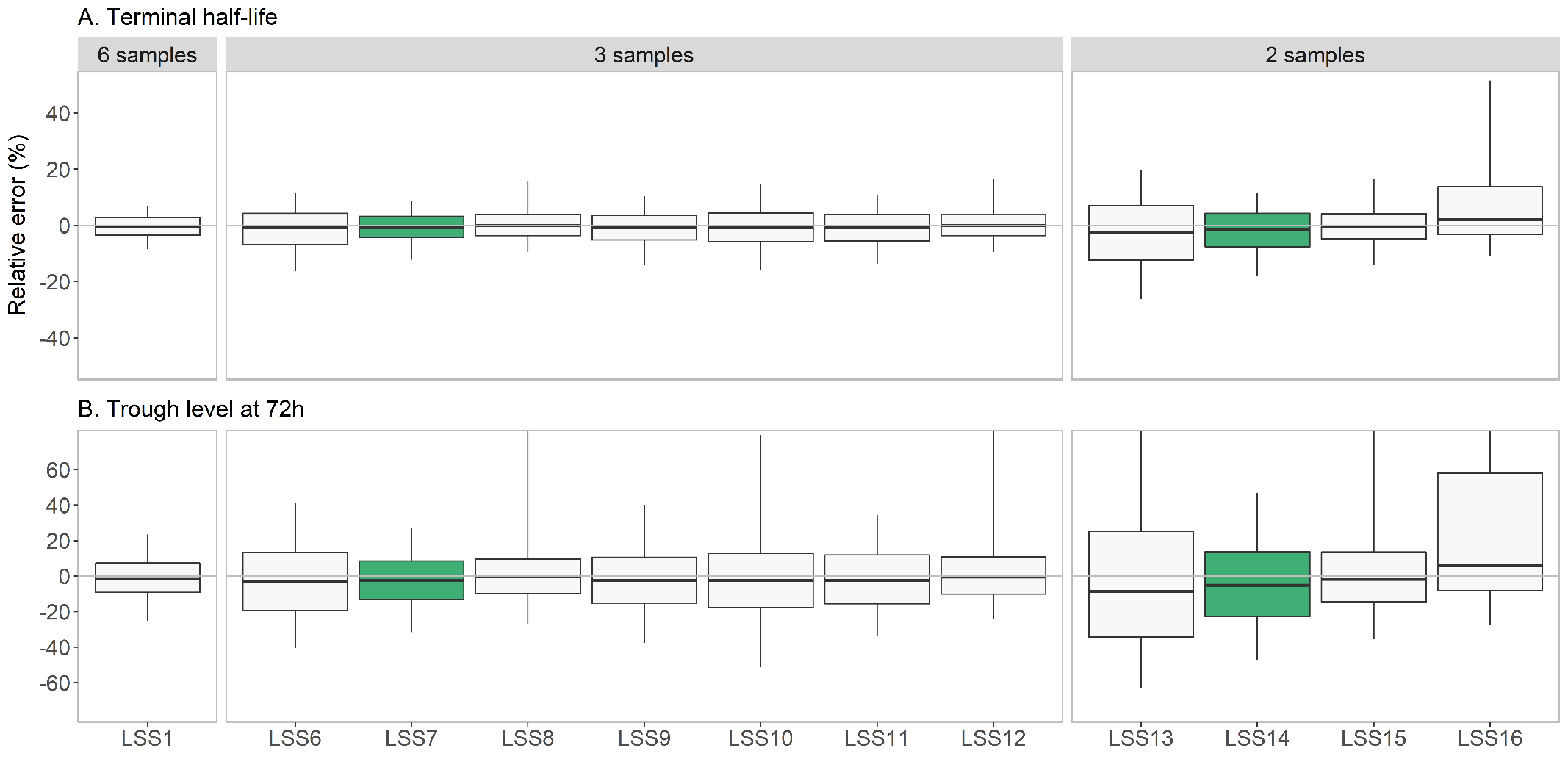

Figure 2: Relative error between the *true* simulated PK parameters and PK parameters estimated by Bayesian analysis for a selection of the evaluated limited sampling strategies (LSS) of BAX 855 of children <12 years.** The parameters elimination half-life (t1/2) and FVIII level at 72h after dose (C72) are presented. The boxes of the boxplots present the median (middle line) and interquartile range (IQR) and the whiskers extend to the 5^th^ and 95^th^ percentile. The green boxes present the preferred limited sampling strategies. For readability, the y-axis of the C72 plots are limited from -75% to 75%, while maintaining all data.

**
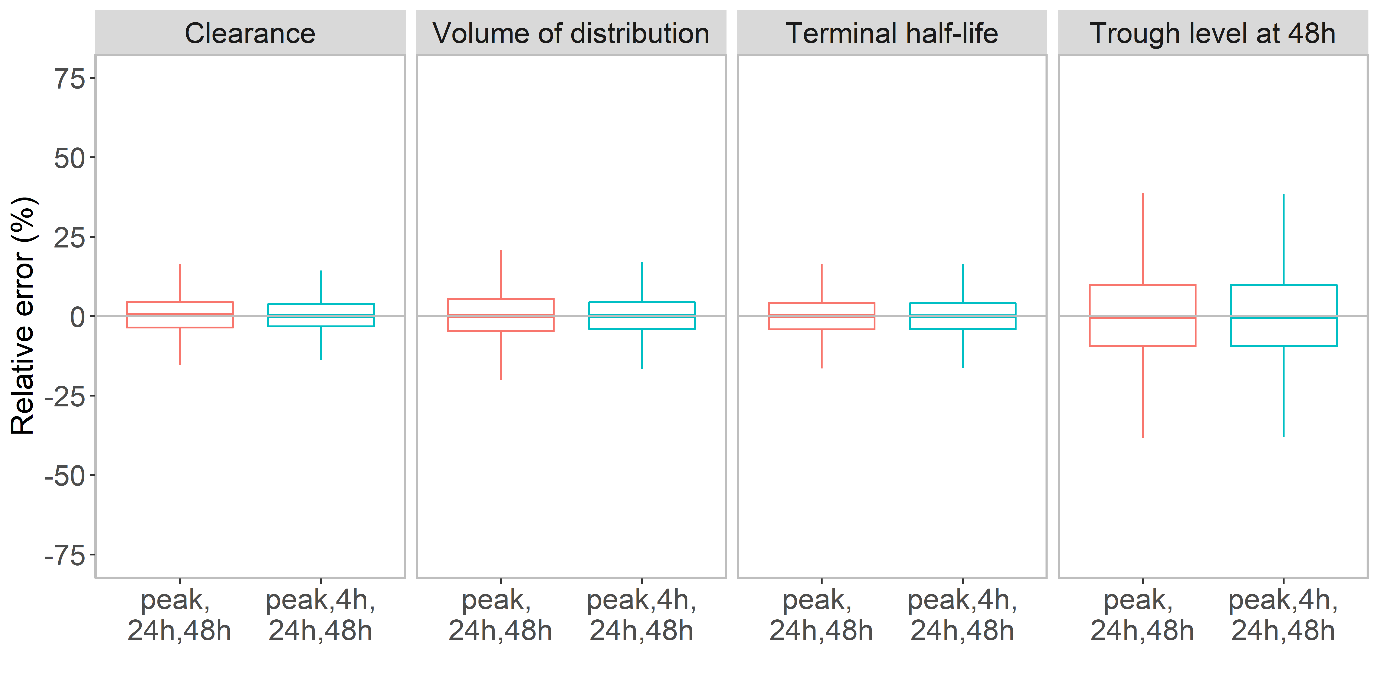
**

**Figure 3: Relative error between the *true* simulated PK parameters and PK parameters estimated by Bayesian analysis for the evaluated limited sampling strategies of FVIII SHL concentrates.** The parameters clearance (CL), steady state volume of distribution (VSS), elimination half-life (t1/2) and FVIII level 48h after the dose (C48) are presented. The boxes of the boxplots present the median (middle line) and interquartile range (IQR) and the whiskers extend to the 5^th^ and 95^th^. The peak was defined as 15-30min after dose. No clinically relevant differences can be seen when the strategy with and without the 4h sample are compared.

|  | Clearance  (CL) | | Steady state distribution volume (VSS) | | Terminal half-life  (t1/2) | | | | Predicted FVIII level at t=72 h  (C72) | |
| --- | --- | --- | --- | --- | --- | --- | --- | --- | --- | --- |
|  | MPE (%) | MAPE (%) | MPE (%) | MAPE (%) | | MPE (%) | MAPE (%) | MPE (%) | | MAPE (%) |
| 6 samples |  |  |  |  | |  |  |  | |  |
| LSS1 | 1.4 (1.0-1.8) | 5.8 (5.5-6.0) | 1.0 (0.4-1.6) | 7.8 (7.4-8.2) | | -0.5 (-0.8--0.3) | 3.9 (3.7-4.0) | -0.7 (-1.7-0.2) | | 11.4 (10.7-12.1) |
| 4 samples |  |  |  |  | |  |  |  | |  |
| LSS2 | 1.8 (1.4-2.3) | 6.5 (6.2-6.8) | 1.1 (0.4-1.7) | 8.6 (8.2-9.0) | | -0.9 (-1.2--0.5) | 4.7 (4.5-4.9) | -1.5 (-2.5--0.4) | | 13.5 (12.8-14.2) |
| LSS3 | 1.9 (1.4-2.3) | 6.7 (6.3-7.0) | 1.2 (0.5-1.8) | 8.6 (8.2-9.0) | | -0.8 (-1.1--0.4) | 4.8 (4.6-5.0) | -1.1 (-2.2-0.1) | | 15.0 (14.2-15.8) |
| LSS4 | 1.8 (1.2-2.3) | 7.0 (6.7-7.4) | 1.4 (0.7-2.0) | 8.6 (8.2-9.0) | | -0.5 (-0.8--0.1) | 4.6 (4.3-4.8) | 1.2 (-0.4-2.8) | | 15.7 (14.3-17.0) |
| LSS5 | 1.7 (1.2-2.2) | 6.9 (6.6-7.2) | 1.3 (0.7-2.0) | 8.9 (9.5-9.3) | | -0.5 (-0.8--0.2) | 4.0 (3.8-4.2) | -0.2 (-1.6-1.3) | | 12.6 (11.4-13.8) |
| 3 samples |  |  |  |  | |  |  |  | |  |
| LSS6 | 2.6 (2.1-3.2) | 7.5 (7.1-7.8) | 1.0 (0.4-1.7) | 8.9 (8.4-9.3) | | -1.4 (-1.9--0.9) | 6.8 (6.5-7.1) | -2.1 (-3.5--0.6) | | 20.1 (19.2-21.1) |
| LSS7 | 2.1 (1.6-2.6) | 7.2 (6.9-7.5) | 1.2 (0.6-1.9) | 8.9 (8.5-9.3) | | -0.9 (-1.3--0.6) | 4.8 (4.6-5.1) | -1.1 (-2.6-0.5) | | 14.9 (13.6-16.2) |
| LSS8 | 0.3 (-0.3-0.9) | 8.2 (7.7-8.6) | 1.5 (0.8-2.2) | 9.0 (8.6-9.5) | | 1.3 (0.8-1.9) | 5.8 (5.3-6.2) | 30.5 (18.6-42.4) | | 42.8 (31.0-54.5) |
| LSS9 | 2.4 (1.8-2.9) | 7.5 (7.1-7.9) | 1.2 (0.5-1.8) | 8.7 (8.3-9.1) | | -1.1 (-1.5--0.7) | 5.7 (5.4-5.9) | -0.1 (-1.8-1.7) | | 18.7 (17.4-20.1) |
| LSS10 | 2.6 (2.0-3.3) | 8.5 (8.1-8.9) | 1.4 (0.8-2.1) | 8.8 (8.4-9.2) | | -0.8 (-1.3--0.3) | 6.9 (6.6-7.3) | 2.9 (0.5-5.2) | | 25.6 (23.8-27.5) |
| LSS11 | 2.1 (1.6-2.6) | 7.1 (6.7-7.4) | 1.2 (0.6-1.9) | 9.0 (8.6-9.5) | | -0.9 (-1.3--0.4) | 5.8 (5.5-6.0) | -0.8 (-2.3-0.7) | | 17.3 (16.1-18.4) |
| LSS12 | 0.3 (-0.3-0.9) | 8.0 (7.6-8.4) | 1.4 (0.8-2.1) | 9.0 (8.6-9.5) | | 1.3 (0.7-1.9) | 5.8 (5.4-6.3) | 29.5 (17.9-41.1) | | 41.7 (30.2-53.2) |
| 2 samples |  |  |  |  | |  |  |  | |  |
| LSS13 | 5.2 (4.4-6.1) | 11.8 (11.2-12.4) | 1.2 (0.5-1.8) | 9.1 (8.6-9.5) | | -2.5 (-3.3--1.7) | 11.3 (10.8-11.8) | 1.0 (-1.9-4.0) | | 38.2 (36.2-40.2) |
| LS14 | 3.6 (3.0-4.3) | 9.0 (8.5-9.4) | 1.2 (0.5-1.9) | 9.0 (8.6-9.5) | | -2.0 (-2.5--1.4) | 7.2 (6.9-7.6) | -2.8 (-4.7--0.9) | | 22.9 (21.5-24.3) |
| LS15 | 1.2 (0.6-1.9) | 8.9 (8.5-9.4) | 1.4 (0.7-2.1) | 9.0 (8.6-9.5) | | 0.6 (0.1-1.2) | 6.9 (6.4-7.4) | 28.7 (16.8-40.6) | | 46.2 (34.5-57.9) |
| LS16 | -4.8 (-5.6--3.9) | 12.1 (11.5-12.7) | 1.8 (1.2-2.5) | 9.1 (8.7-9.6) | | 8.8 (7.6-10.0) | 13.1 (12.0-14.1) | 141.1 (103.9-178.3) | | 152.4 (115.4-189.5) |

**Table 1: Bias and inaccuracy of evaluated parameters and examined limited sampling strategies for children <12 years,** represented by the mean percentage error (MPE) and mean absolute percentage error (MAPE).
